# Impaired olfactory function in bipolar disorder patients during acute episodes regardless of psychotic symptoms

**DOI:** 10.3389/fpsyt.2023.1284567

**Published:** 2023-10-30

**Authors:** Yingying Li, Huiqian Yuan, Xianlin Liu, Langjun Su, Chunhong Yang, Chao Chen, Chunyang Li

**Affiliations:** Department of Psychiatry, Shunde WuZhongpei Memorial Hospital, Foshan, China

**Keywords:** bipolar disorder, psychotic symptoms, olfactory identification, olfactory sensitivity, acute episodes, euthymic episodes

## Abstract

**Background:**

The aim of this study was to analyze whether the presence of psychotic symptoms affects olfactory function in patients with bipolar disorder (BD). We also compared olfactory function between the period of episode and remission in patients with BD.

**Methods:**

BD patients in the acute phase were tracked to the remission stage. The psychiatric symptoms and social function of the enrolled subjects were assessed using the Hamilton Rating Scale for Depression (HAMD), the Young Mania Rating Scale (YMRS), the Hamilton Rating Scale for Anxiety (HAMA), the Positive and Negative Syndrome Scale (PANSS) and the Global Assessment Function (GAF). Olfactory sensitivity (OS) and olfactory identification (OI) was assessed using the Sniffin’ Sticks test. Differences in OS and OI among the episodic group, the euthymic group, and the healthy control (HC) group were compared. According to whether BD is accompanied by psychotic symptoms, the OS and OI in the BD with psychotic symptoms group (P-BD), the BD without psychotic symptoms group (NP-BD), and the HC group were compared.

**Results:**

The P-BD and NP-BD groups exhibited impaired OI compared with the HC group, but there was no significant difference in OI between the P-BD and NP-BD groups, or in OS among all three groups. All patients with episodic BD had significantly lower OS and OI compared with the HC group. OI in euthymic BD patients was still impaired; however, OS recovered, showing no significant difference compared with that in the HC group.

**Conclusion:**

The results indicate that patients with episodic BD have impaired OS and OI, regardless of psychotic symptoms. OI may be a characteristic marker of BD, and OS may be a state marker that can be used to distinguish between episodic and euthymic BD.

## 1. Introduction

Bipolar disorder (BD) is a major mental disorder characterized by recurrent episodes of high or low mood, alternating depressive or manic or hypomanic episodes with remission. It is characterized by a high recurrence rate, high misdiagnosis rate, high disability rate, and high suicide risk (1). In recent years, researchers in the field of psychiatry have focused on searching for evidence that can help in the diagnosis of mental illness. It has been reported that olfactory disorders can be used as markers of a series of neuropsychiatric diseases, and the evaluation of olfactory function involves measures of olfactory identification (OI), olfactory sensitivity (OS), and other factors (2). Recent evidence suggests that many patients with obsessive-compulsive disorder, schizophrenia, depression, BD, post-traumatic stress disorder and other diseases have abnormal olfactory function (3–6). Lahera et al. (7) and Cumming et al. (8) reported that many BD patients have OI defects. However, Swiecicki et al. (9) reported conflicting results in a study that compared the olfactory function of 21 bipolar depression patients with 30 HC groups. The results revealed no significant differences in OS and OI between the two groups (9). To date, few relevant studies have examined olfactory function in BD, and the results have been inconsistent. However, most of these studies have supported the existence of olfactory dysfunction in BD patients.

Some studies have reported that patients with P-BD and NP-BD have different clinical characteristics (10, 11). P-BD is reported to be associated with a wide range of brain structural abnormalities, whereas NP-BD is reported to be manifested as defects in localized cerebral cortical regions (12). The pattern of gray matter volume loss in patients with P-BD has been suggested to resemble, at least in part, the brain structural abnormalities observed in patients with schizophrenia (13). Compared with NP-BD, P-BD also involves significant changes in neurobiochemistry, event-related potentials, brain imaging, genetics, and other features (14). Kamath et al. (15) specifically pointed out that the OI functional defect occurred only in P-BD patients. However, several studies on olfactory function in BD patients revealed that OI in BD patients was not affected by psychotic symptoms (8, 16). However, the results of previous studies are contradictory. The current clinical diagnosis of BD is based on intact medical history collection and psychiatric examination, which are highly subjective measures that lack objective biological indicators, and emotional symptoms are often masked by other symptoms. Therefore, the establishment of research that is based on biological markers for the diagnosis of BD, especially for the differential diagnosis of other diseases such as P-BD and schizophrenia, is extremely important (17).

In the study of olfactory function in patients with BD, some researchers have pointed out that OI is a trait marker of BD but has no specificity, whereas OS is a state marker that can be used to distinguish between the episodic and euthymic BD (4). Kazour et al. (18, 19) reported that OI, which involves the recognition of pleasant odor, is a trait marker for BD patients. However, the above conclusions are still controversial (16, 20). Thus, differing interpretations have been reported regarding the olfactory function during episodic and euthymic BD in previous research. Next, we will carry out further research to clarify this issue.

Many existing studies have reported that mental disorders are closely related to olfactory function, but few studies have specifically examined olfactory function in BD, and the results have been inconsistent. In particular, few studies have directly examined the relationship between olfactory function and P-BD and NP-BD, and the sample sizes of existing studies are small. Additionally, there is a lack of direct longitudinal comparison of olfactory function between episodic and euthymic BD. On the basis of evidence that the occurrence of psychotic symptoms can increase the difficulty of treatment, olfactory impairment and poor clinical prognosis of BD patients, in the current study we compared whether there were differences in olfactory function between P-BD and NP-BD to explore whether olfactory disorders could provide a screening index for early identification of P-BD. Moreover, we conducted a longitudinal comparison of olfactory function between episode and remission in BD patients with and without psychotic symptoms, providing new aids for evaluating clinical efficacy and improving clinical prognosis in BD patients.

## 2. Materials and methods

### 2.1. Participants

For the case group, we selected all BD patients who were hospitalized at Shunde WuZhongpei Memorial Hospital, Foshan City from August 2022 to November 2022. A total of 74 patients with episodic BD who met the criteria were enrolled, and 52 cases were followed up until remission. According to whether BD was accompanied by psychotic symptoms, the case groups were divided into a P-BD group and an NP-BD group. Among them, 35 cases were enrolled in the episodic P-BD group, and 20 cases were followed up in the euthymic group. Thirty nine cases were enrolled in the episodic NP-BD group, and 32 cases were followed up in the euthymic group. In addition, 44 hospital staff matched with the case group for sex, age, years of education and other general data were selected as the HC group. The researchers put out recruitment announcements for volunteers in the hospital, and the participants volunteered to sign up. This study was approved by the Ethics Committee of Shunde WuZhongpei Memorial Hospital, Foshan City. All participants signed a written informed consent form before enrollment.

### 2.2. Criteria

The inclusion criteria for episodic BD were used to select patients who met each of the following requirements at the same time: meeting the criteria for the diagnosis of BD in the Diagnostic and Statistical Manual of Mental Disorders, Fifth Edition (DSM-5); aged 18–60 years old; no other mental disorders; primary school education or above; Han nationality; HAMD ≥20 or YMRS ≥20 at enrollment.

The inclusion criteria for euthymic BD were used to select patients who met each of the following requirements at the same time: no obvious clinical symptoms; stable condition for more than 1 month; HAMD score ≤ 7; YMRS score ≤ 7.

The enrollment criteria for the HC group were used to select participants who met each of the following requirements at the same time: absence of psychiatric illness; first-degree relatives had no history of major mental illness; HAMD score ≤ 7; YMRS score ≤ 7; age 18–60 years old; primary school education or above; Han nationality.

Participants were not included if any of the following exclusion criteria were met: history of head, neck trauma and nasal surgery, and history of neurological disorders; intellectual disability (IQ less than 70); infection with respiratory diseases within 2 weeks or have any diseases that may affect olfactory function (such as nasal polyps, common cold, or allergies); a history of alcohol, drug abuse or dependence in the past year; inability to cooperate with the completion of the olfactory examination; pregnant or lactating women; presence of heart, liver and kidney diseases, malignant tumors, or other serious physical diseases.

### 2.3. Methods

We designed a questionnaire to collect the age, sex, physical health and other basic demographic and sociological data of the enrolled subjects, and to collect and record the clinical data such as diagnosis, disease course, age of onset, and medication in the case group.

The Hamilton Rating Scale for Depression (HAMD) is mainly used to reflect the severity of depressive symptoms. A five-level scoring method ranging from 0 to 4 points is adopted. The assessment was generally conducted in the week prior to the assessment.

The Young Mania Rating Scale (YMRS) is mainly used to assess the presence or absence of manic symptoms and the severity of symptoms. The YMRS contains 11 items in total, most of which are focused on evaluating the situation in the last week. Scores of 0–5 points indicate no obvious symptoms of mania, scores of 6–12 points are classified as mild, scores of 13–19 points are classified as moderate, scores of 20–29 points are classified as severe, and scores of more than 30 points are classified as extremely severe.

The Hamilton Rating Scale for Anxiety (HAMA) is mainly used to assess the severity of anxiety symptoms. The HAMA contains 14 items in total. Each item ranges from 0 to 4 points, providing a general evaluation of the situation during the latest week.

The Positive and Negative Syndrome Scale (PANSS) comprises 30 items, which are graded from 1 to 7. The PANSS is mainly used to assess the severity of psychiatric symptoms.

The Global Assessment Function (GAF) measure is used to comprehensively assess the psychological, social, and occupational functioning of participants. There are 100 grades in the GAF, with scores ranging from 1 to 100 points. The lower the score, the more severe the impairment of function.

To conduct olfactory function assessment, the Sniffin’ Sticks test (SST) included the OI and OS tests. The OI test consisted of 16 groups with one olfactory stick in each group. The following 16 familiar odors were presented to each subject: orange, leather, chocolate, mint, etc. The experimenters opened each stick in turn, and placed it 1 ~ 2 cm below the nostrils of the subjects for about 3 s, and the subjects were asked to identify the smell of the stick by choosing one of four possible answers. Each correct response was scored as 1 point. The total score ranged from 0 to 16 points, with higher scores indicating better OI ability. The OS test was conducted in a total of 16 groups, with three olfactory sticks in one group. The highest concentration of olfactory agent was 4% n-butanol, and the multiple ratio dilution was 1–16 grades. The lower the concentration grade, the higher the recorded score. After familiarizing themselves with the smell of the highest concentration n-butanol, the participant began the OS test. Each group was tested with one olfactory stick containing n-butanol, and the other two sticks were used as blank controls. After two correct answers in the test at the same concentration, one concentration was decreased until the subject could not smell it, and the level was recorded. The concentration was then increased step by step until the n-butanol sniffing stick could be correctly sniffed and the grade recorded. After a series of 7 switches between concentrations, the geometric mean of the last four staircase reversal points was used as the score of the OS. The higher the score, the better the OS (21).

Before the test, all participants completed the personal general condition questionnaire, and were screened by two professional psychiatrists with the HAMD, YMRS, HAMA, PANSS, and other scales. The olfactory test was performed after the screening criteria were met. The episodic BD group was followed up until symptoms were relieved and the second olfactory test and scale evaluation were completed after the inclusion criteria for the remission period were met. The HC group completed only one olfactory test and scale assessment at enrollment. The test was conducted in a quiet, separate, air-circulating indoor room. The whole process of olfactory testing and scale evaluation took 80–100 min.

### 2.4. Statistical analysis

SPSS25.0 software was used for statistical analysis. The mean ± standard deviation was used to represent the normally distributed data, and the median (lower quartile, upper quartile) was used to represent the non-normally distributed data. For the comparison between two means, the independent sample t-test and variance homogeneity test were used for normally distributed data, and a non-parametric test was used for non-normally distributed data. For the comparison between the means of multiple groups, the normality data were analyzed using one-way analysis of variance, the difference was statistically significant, and pairwise comparisons were carried out. A non-parametric testing method was used for non-normal data, and if the difference was statistically significant, pairwise comparisons were further conducted, and the Bonferroni method was used to correct the test level α value. Qualitative data were expressed by case number and component ratio, the Chi-square test was used for comparison between groups, and the Wilcoxon signed rank test was used for paired data. Multiple linear regression analysis was used to control factors. Correlation analysis was conducted using Pearson’s or Spearman’s methods. The test level was *α* = 0.05.

## 3. Results

### 3.1. Comparison among episodic BD patients

#### 3.1.1. General information and clinical features

In the current study, a total of 74 episodic BD patients and 44 HCs were examined. The former could be divided into a P-BD group with 35 cases and an NP-BD group with 39 cases according to whether BD was accompanied by psychotic symptoms. We found no significant differences in age, sex, smoking and education level among all groups (*p* > 0.05). Additionally, there were no significant differences in the course of disease and age of first onset between the P-BD and NP-BD groups (*p* > 0.05) (Table 1).

**Table 1:** Sociodemographics, clinical characteristics and olfactory function among P-BD, NP-BD, and HC groups during acute episodes.

Variable	P-BD group (*N* = 35)	NP-BD group (*N* = 39)	HC group (*N* = 44)	*t/*χ^2^	*p*
**Sociodemographic**					
Age (years)	39 (29, 52)	35 (30, 42)	43 (35.3,47.8)	5.050	0.080
Sex (M/F)	17/18	23/16	19/25	2.103	0.349
Smoking, N (%)	9 (25.7)	10 (25.6)	6 (13.6)	2.395	0.302
Years of education	9 (7, 12)	11 (9, 14)	12 (8, 15)	3.678	0.159
**Clinical characteristics**					
Course of disease (years)	10 (6, 20)	11 (6, 17)	–	−0.315	0.753
Age of onset (years)	27.1 ± 10.0	23.5 ± 7.2	–	7.052	0.074
HAMD	8 (3, 20)	6 (3, 14)	2 (1, 3.8)	–	–
YMRS	23 (13, 28)	24 (20, 32)	0 (0, 1.8)	–	–
HAMA	6 (3, 12)	4 (2, 8)	1 (0.3, 3)	–	–
GAF	35 (32, 45)	44 (35, 46)	94.5 (92, 95)	–	–
PANSS	67 (61, 72)	55 (49, 65)	30 (30, 31)	–	–
**Medications^A^, N (%)**
	Atypical antipsychotics	35 (100.0)	37 (94.9)	–	1.845	0.174
	Risperidone	10 (28.6)	8 (20.5)	–	0.651	0.420
	Olanzapine	9 (25.7)	8 (20.5)	–	0.282	0.595
	Quetiapine	9 (25.7)	16 (41.0)	–	1.933	0.164
	Aripiprazole	3 (8.6)	4 (10.3)	–	0.061	0.805
Mood stabilizers	35 (100.0)	39 (100.0)	–	–	–
Lithium	25 (71.4)	30 (76.9)	–	0.292	0.589
Valproate	22 (62.9)	25 (64.1)	–	0.012	0.912
Oxcarbazepine	4 (11.4)	0 (0)	–	–	–
Lamotrigine	2 (5.7)	0 (0)	–	–	–
Antidepressants	4 (11.4)	4 (10.3)	–	0.026	0.871
Benzodiazepines	26 (74.3)	18 (46.2)	–	6.056	0.014*
**Olfactory function**					
OS	5 (4, 8)	5.5 (4, 8)	7 (5.5, 8.4)	4.216	0.121
OI	10 (8, 13)	12 (10, 13)	13.5 (12.3, 14)	26.217	0.000*

HAMD, Hamilton Rating Scale for Depression; YMRS, Young Mania Rating Scale; HAMA, Hamilton Rating Scale for Anxiety; GAF, Global Assessment Function; PANSS, Positive and Negative Syndrome Scale; P-BD, psychotic BD; NP-BD, non-psychotic BD; HC, healthy control; OS, olfactory sensitivity; OI, olfactory identification. ^A^Patients may have received more than one medication type. *p < 0.05.

#### 3.1.2. Comparison of OS and OI among groups

**Figure 1:**
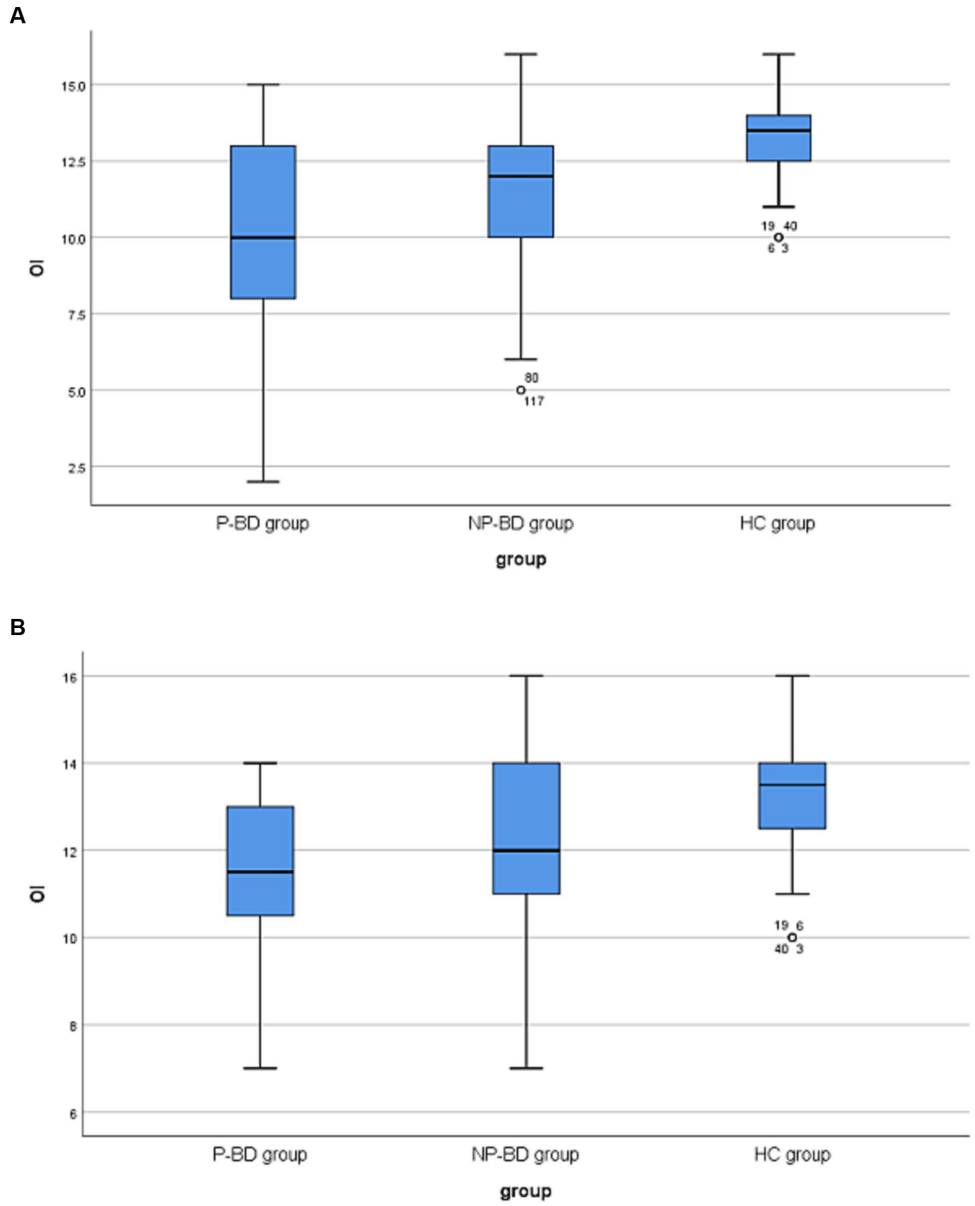
**(A)** Boxplots of OI among P-BD, NP-BD and HC group during acute episodes. **(B)** Boxplots of OI among P-BD, NP-BD and HC group during euthymia. OI, olfactory identification; P-BD, psychotic BD; NP-BD, non-psychotic BD; HC, healthy control.

The results revealed that there was no significant difference in OS among episodic P-BD patients and episodic NP-BD patients and the HC group (*χ*^2^ = 4.216, *p* = 0.121). The OI of episodic P-BD patients, episodic NP-BD patients and the HC group was compared, and there were significant differences among all groups (*χ*^2^ = 26.217, *p* < 0.001). Further pairwise comparison was conducted, and the Bonferroni method was used to adjust the test level (*α* = 0.0167). Additionally, the results showed that the difference between the P-BD, NP-BD, and HC groups was statistically significant (*Z* = −4.612, *p* < 0.001; *Z* = −3.851, *p* < 0.001), whereas the difference between P-BD and NP-BD groups was not statistically significant (*Z* = −1.647, *p* = 0.100). Therefore, it can be inferred that the OI of the episodic P-BD patients and the episodic NP-BD patients was significantly lower compared with that in the HC group (Table 1 and Figure 1).

#### 3.1.3. Correlation analysis of olfactory function

The correlation between OS and OI in episodic P-BD patients and episodic NP-BD patients was analyzed for the general data and clinical features. The results revealed that OI was negatively correlated with the course of the disease in patients with episodic P-BD (*r* = −0.356, *p* = 0.036). OS was negatively correlated with PANSS score (*r* = −0.358, *p* = 0.025), age (*r* = −0.333, *p* = 0.038), and course of disease (*r* = −0.317, *p* = 0.049), and OI was positively correlated with years of education in episodic NP-BD patients (*r* = 0.548, *p* < 0.001). The OI of NP-BD patients was affected by sex (*r* = 0.354, *p* = 0.027). Correlation analysis of other items did not reveal statistically significant associations (Tables 2 3).

**Table 2:** Correlation of olfactory function in the P-BD group and NP-BD group with general data and clinical features during acute episodes and euthymia.

		Age		Course of disease		Years of education		Age of onset		Sex	
		*r*	*p*	*r*	*p*	*r*	*p*	*r*	*p*	*r*	*p*
Episodic											
P-BD	OS	−0.009	0.958	−0.077	0.659	0.129	0.462	0.213	0.218	−0.057	0.746
	OI	−0.317	0.064	−0.356	0.036*	0.300	0.080	0.062	0.721	0.148	0.396
NP-BD	OS	−0.333	0.038*	−0.317	0.049*	0.223	0.173	−0.106	0.519	−0.107	0.517
	OI	−0.045	0.784	−0.242	0.139	0.548	0.000*	0.171	0.297	0.354	0.027*
Euthymic											
P-BD	OS	−0.084	0.725	−0.050	0.835	0.028	0.908	−0.070	0.771	−0.420	0.065
	OI	−0.641	0.002*	−0.422	0.064	0.383	0.096	−0.476	0.034*	−0.106	0.657
NP-BD	OS	−0.052	0.778	−0.188	0.302	0.387	0.029*	0.091	0.620	−0.173	0.344
	OI	0.278	0.124	−0.022	0.906	0.443	0.011*	0.353	0.048*	0.474	0.006*

P-BD, psychotic BD; NP-BD, non-psychotic BD; OS, olfactory sensitivity; OI, olfactory identification. *p < 0.05.

**Table 3:** Correlation of olfactory function in the P-BD group and NP-BD group with emotional symptoms and social function during acute episodes and euthymia.

		HAMD		YMRS		HAMA		GAF		PANSS	
		*r*	*p*	*r*	*p*	*r*	*p*	*r*	*p*	*r*	*p*
Episodic											
P-BD	OS	−0.176	0.311	−0.153	0.381	−0.303	0.077	−0.020	0.909	−0.279	0.104
	OI	0.144	0.409	−0.058	0.743	0.103	0.554	0.251	0.146	−0.253	0.142
NP-BD	OS	−0.230	0.159	0.100	0.545	−0.214	0.190	0.250	0.125	−0.358	0.025*
	OI	0.190	0.247	−0.049	0.768	0.186	0.256	−0.080	0.630	−0.167	0.309
Euthymic											
P-BD	OS	−0.032	0.892	−0.047	0.844	−0.112	0.640	−0.107	0.652	−0.108	0.651
	OI	0.188	0.427	0.122	0.607	0.154	0.517	−0.056	0.814	0.028	0.908
NP-BD	OS	−0.362	0.042*	0.069	0.708	−0.248	0.172	0.346	0.052	−0.248	0.170
	OI	−0.147	0.421	−0.264	0.144	0.127	0.489	0.125	0.494	−0.129	0.483

HAMD, Hamilton Rating Scale for Depression; YMRS, Young Mania Rating Scale; HAMA, Hamilton Rating Scale for Anxiety; GAF, Global Assessment Function; PANSS, Positive and Negative Syndrome Scale; P-BD, psychotic BD. NP-BD, non-psychotic BD; OS, olfactory sensitivity; OI, olfactory identification. *p < 0.05.

### 3.2. Comparison between euthymic BD patients and the HC group

#### 3.2.1. General information and clinical features

The disease condition and olfactory function of P-BD and NP-BD patients were followed up. At the end of the follow-up, a total of 52 patients were enrolled in the remission group, and the remission rate reached 70.27%. In 11 patients, the disease had not been controlled, and the resulting shedding rate was 14.86%. In addition, 11 patients dropped out during the outpatient follow-up stage, resulting in a shedding rate of 14.86%.

There were 52 euthymic BD patients, and patients could be divided into 20 cases in the P-BD group and 32 cases in the NP-BD group according to whether they exhibited psychotic symptoms at the time of enrollment. There were 44 cases in the HC group. There were no statistically significant differences in sex, smoking status, or education level among all groups. There were no significant differences in age of onset or disease course between P-BD and NP-BD groups (*p* > 0.05), but there were significant differences in age among the three groups (*t* = 3.902, *p* = 0.024) (Table 4).

**Table 4:** Sociodemographics, clinical characteristics and olfactory function among euthymic P-BD, NP-BD, and HC groups.

Variable	P-BD group (*N* = 20)	NP-BD group (*N* = 32)	HC group (*N* = 44)	*t/χ* ^2^	*p*
**Sociodemographic**					
Age (years)	36.5 ± 10.6	35.6 ± 8.7	41.1 ± 8.5	3.902	0.024*
Sex (M/F)	10/10	19/13	19/25	1.943	0.378
Smoking, N (%)	6 (30.0)	8 (25.0)	6 (13.6)	2.738	0.254
Years of education	11 (9, 14.3)	11.3 (9, 14)	12 (8, 15)	0.542	0.763
**Clinical characteristics**					
Course of disease (years)	8.8 ± 5.5	12.1 ± 7.3	–	1.938	0.085
Age of onset (years)	27.7 ± 9.4	23.5 ± 7.3	–	3.841	0.079
HAMD	3 (1, 4)	2 (1, 4)	2 (1, 3.8)	–	–
YMRS	4.5 (1.3, 6)	2 (0, 4)	0 (0, 1.8)	–	–
HAMA	2 (1, 5)	1.5 (1, 3)	1 (0.3, 3)	–	–
GAF	79.5 (69, 85)	81 (75, 85)	94.5 (92, 95)	–	–
PANSS	38.5 (34.3,42.8)	34 (31.3,37.8)	30 (30, 31)	–	–
**Medications^A^, N (%)**					
Atypical antipsychotics	19 (95.0)	30 (93.8)	–	0.035	1.000
Risperidone	7 (35.0)	8 (2.1)	–	0.600	0.439
Olanzapine	5 (25.0)	9 (28.1)	–	0.061	0.805
Quetiapine	6 (30.0)	9 (28.1)	–	0.021	0.885
Aripiprazole	3 (15.0)	4 (12.5)	–	0.066	1.000
Mood stabilizers	20 (100.0)	32 (100.0)	–	–	–
Lithium	17 (85.0)	23 (71.9)	–	1.194	0.330
Valproate	14 (70.0)	23 (71.9)	–	0.021	0.885
Oxcarbazepine	2 (10.0)	2 (6.3)	–	–	–
Lamotrigine	2 (10.0)	0 (0.0)	–	–	–
Antidepressants	1 (5.0)	10 (31.3)	–	–	–
Benzodiazepines	15 (75.0)	11 (34.4)	–	0.125	0.004*
**Olfactory function**					
OS	8.3 (6.6, 8.9)	7 (5.6, 8)	7 (5.5, 8.4)	3.430	0.180
OI	11.5 (10.3, 13)	12 (11, 14)	13.5 (12.3,14)	12.699	0.002*

HAMD, Hamilton Rating Scale for Depression; YMRS, Young Mania Rating Scale; HAMA, Hamilton Rating Scale for Anxiety; GAF, Global Assessment Function; PANSS, Positive and Negative Syndrome Scale; P-BD, psychotic BD; NP-BD, non-psychotic BD; HC, healthy control; OS, olfactory sensitivity; OI, olfactory identification. ^A^Patients may have received more than one medication type. *p < 0.05.

#### 3.2.2. Comparison of OS and OI among groups

The results revealed that there was no significant difference in OS among the euthymic P-BD group, euthymic NP-BD group, and HC group (*χ*^2^ = 3.430, *p* = 0.180). After controlling for age using multiple linear regression analysis, the results still showed no significant differences in OS among the three groups (Statistical value = −0.896, *p* = 0.372). The OI of the euthymic P-BD group, euthymic NP-BD group, and HC group were compared, and the results revealed statistically significant differences among all groups (*χ*^2^ = 12.699, *p* = 0.002). After controlling for age as a confounding factor, there was still a statistically significant difference in OI among the three groups (Statistical value = 3.833, *p* < 0.001). The data were combined with the results of the pairwise comparison, and the test level was adjusted using the Bonferroni method (*α* = 0.0167). The results showed that the OI of euthymic P-BD patients and euthymic NP-BD patients was worse than that in the HC group. However, there was no statistically significant difference in OI between the euthymic P-BD group and the euthymic NP-BD group (Table 4 and Figure 1).

#### 3.2.3. Correlation analysis of olfactory function

The correlation analysis showed that OI of euthymic P-BD was negatively correlated with age and age of first onset (*r* = −0.641, *p* = 0.002; *r* = −0.476, *p* = 0.034). OS in euthymic NP-BD was positively correlated with years of education and negatively correlated with HAMD score (*r* = 0.387, *p* = 0.029; *r* = −0.362, *p* = 0.042), and OI was positively correlated with age of first onset and years of education (*r* = 0.353, *p* = 0.048; *r* = 0.443, *p* = 0.011). The OI of patients with NP-BD was affected by sex (*r* = 0.474, *p* = 0.006). Correlation analysis of other items did not reveal statistically significant associations (Tables 2 3).

### 3.3. Comparison between the total episodic BD group and the HC group

There were no significant differences in age, sex, smoking, and education level between the episodic BD group (*N* = 74) and the HC group (*N* = 44) (*p* > 0.05). The OS and OI of episodic BD group were significantly lower than that of the HC group (Table 5).

**Table 5:** Comparison of the olfactory function of episodic and euthymic patients among different groups.

Project	OS	OI
All patients with episodic BD (*N* = 74)	5.5 (4, 8)	11 (9, 13)
HC group (*N* = 44)	7 (5.5, 8.4)	13.5 (12.3, 14)
*Z*	−1.997	−4.937
*P*	0.046*	<0.001*
All patients with euthymic BD (*N* = 52)	7 (6, 8.5)	12 (11, 13.8)
HC group (*N* = 44)	7 (5.5, 8.4)	13.5 (12.3, 14)
*Z*	−0.830	−3.405
*P*	0.406	0.001*
All patients with euthymic BD—		
All patients with episodic BD		
*Z*	−4.126	−1.245
*P*	< 0.001*	0.213
Episodic P-BD group—		
Euthymic P-BD group		
*Z*	−4.888	−0.707
*P*	< 0.001*	0.479
Euthymic NP-BD group—		
Episodic NP-BD group		
*Z*	−2.645	−1.035
*P*	0.008*	0.301

BD, bipolar disorder; P-BD, psychotic BD; NP-BD, non-psychotic BD; OS, olfactory sensitivity; OI, olfactory identification. *p < 0.05.

The correlation analysis showed that the OS and OI of patients in the total episodic group were negatively correlated with the course of the disease (*r* = −0.258, *p* = 0.027; *r* = −0.277, *p* = 0.017). OI was positively correlated with years of education (*r* = 0.416, *p* < 0.001), OS was negatively correlated with HAMA score and PANSS score (*r* = −0.249, *p* = 0.032; *r* = −0.335, *p* = 0.004). Correlation analysis between the other items revealed no statistically significant associations.

### 3.4. Comparison between the total euthymic BD group and HC group

There were no significant differences in sex, smoking status, and education level between the total euthymic BD group (*N* = 52) and the HC group (*N* = 44) (*p* > 0.05). The OI of euthymic BD patients was significantly lower than that of the HC group (*p* = 0.001), but there was no significant difference in OS between the euthymic BD group and the HC group (*p* > 0.05) (Table 5).

The correlation analysis revealed that OI of the euthymic BD patients was positively correlated with the years of education (*r* = 0.420, *p* = 0.002). However, no statistically significant associations were found in other correlation analyses (*p* > 0.05).

### 3.5. Comparison between total episodic BD group and total euthymic BD group

The longitudinal comparison of OS and OI in total episodic BD group and total euthymic BD group showed that the difference in OS between the two groups was statistically significant (*Z* = −4.126, *p* < 0.001), but the difference in OI between the two groups was not statistically significant (*p* > 0.05). The results showed that when BD patients were followed up from the episodic stage to the euthymic stage, OS increased significantly, but OI did not change significantly.

The longitudinal dynamic changes of olfactory function were analyzed according to different groups of P-BD and NP-BD. Finally, the results revealed significant differences in OS between the episodic period and the euthymic period in the P-BD and NP-BD groups. The results showed that OS in the remission period of the P-BD and NP-BD groups was significantly higher compared with that in the episodic period, but there was no statistically significant difference in OI between the two groups (*p* > 0.05) (Table 5).

## 4. Discussion

In view of the high structural overlap between the olfactory functional area and the emotional processing brain area, many studies have shown that olfactory function and emotion are related. At present, it has been confirmed that patients with bipolar disorder may have olfactory dysfunction, but the results are controversial. Most studies support that olfactory function can be used as a state marker of bipolar disorder, providing a new tool for the evaluation of efficacy. There are also research hints that olfactory function may be used as an assessment tool for early identification of bipolar disorder, or for distinguishing bipolar disorder with and without psychotic symptoms. This study explored whether psychotic symptoms had an impact on the olfactory function of BD patients, and we compared the olfactory function during acute onset and remission in BD patients with and without psychotic symptoms.

### 4.1. Effect of psychotic symptoms on olfactory function in BD

The results of our study showed that the OI values of the episodic and euthymic periods of P-BD and NP-BD were significantly lower than those in the HC group, and there were no statistically significant differences in OS among the three groups. There was also no significant difference in OI and OS between patients with P-BD and NP-BD, indicating that the presence of psychotic symptoms had no effect on olfactory function in patients with BD.

The findings of several previous reports are in accordance with the present results. Hardy et al. (16) used the University of Pennsylvania Smell Identification Test (UPSIT) method with euthymic BD patients and HC. The results revealed no significant difference in OI between BD patients and HC, and OI was not associated with clinical features (16). Cumming et al. (8) used the UPSIT method to compare the olfactory function of BD patients with and without psychotic symptoms (a total of 20 cases). The results revealed no significant difference in OI between P-BD and NP-BD (8). Brewer et al. (22) conducted olfactory tests on patients with first-onset psychosis and ultimately found no correlation between OI function and untreated psychotic symptoms. Takahashi et al. (23) examined the morphology of olfactory sulci of 26 patients with bipolar I disorder and 24 HC using magnetic resonance imaging. The results showed that the bilateral olfactory sulci in BD patients were significantly shallower compared with those in the HC group, and the measurement of olfactory sulci in BD patients was not significantly correlated with clinical variables such as psychotic symptoms.

However, the findings of some research reports conflict with the results of the current study (15, 24). Possible reasons for this inconsistency include the heterogeneity of the case groups in different studies, the use of different olfactory assessment tools, and the use of different psychotropic drugs.

### 4.2. Comparison of olfactory function among three groups

The current study was a prospective study comparing olfactory function among an episodic BD group, a euthymic BD group, and an HC group. The results of our study showed that OS and OI were impaired in episodic BD patients compared with the HC group, suggesting that OS and OI disorders may become biological markers for early recognition of BD. This is consistent with previous research findings. Zarate et al. (25) compared the olfactory function of patients with bipolar manic episodes and HCs, and found that OI levels of patients with bipolar manic episodes decreased compared with HCs. However, the findings of other studies differ from the results of the current study. Swiecicki et al. (9) compared the olfactory function of 21 episodic bipolar depression patients with 30 HC, and found that there was no significant difference in either OS or OI between the two groups. These results are inconsistent with the current findings, possibly because of the different inclusion criteria for episodic BD patients. Swiecicki et al. only conducted olfactory tests on patients with bipolar depression and the HAMD score of the enrolled patients was 14.1 ± 1.0, while the inclusion criteria for episodic BD depressive patients in our study required a HAMD score of ≥20.

The current study revealed that, compared with the HC group, euthymic BD patients exhibited obvious OI impairment, while there was no significant difference in OS. These findings may indicate that OS of BD patients recovered with the improvement of the disease. This is consistent with the conclusions of Lahera et al. (7). However, Negoias et al. (20) compared the olfactory ability of euthymic BD patients and HCs, and found that there was no significant difference between euthymic BD patients and HCs in terms of OS or OI. The reason for the inconsistency in the results may be related to the small sample size of the enrolled cases in Negoias et al.’s study (21 euthymic BD patients), and the different inclusion criteria used (20). Negoias et al. (20) included euthymic BD patients and required no recurrence of symptoms of depression, hypomania/mania or mixed mania for at least 6 months, whereas the euthymic BD patients in our study were required to be stable for more than 1 month. Hardy et al. (16) compared olfactory function between 20 BD patients in remission (including 12 depressive episodes, five manic episodes, two hypomanic episodes, one mixed episode) and 44 HCs, and reported results similar to those of Negoias et al. (20). Possible reasons for the difference between the findings of Hardy et al.’s study and our study are as follows: first, the sample size of Hardy et al.’s study was small (20 cases) (16). In addition, more female patients were recruited, accounting for 75% of the patient group in Hardy et al.’s study (16). However, in our study, females accounted for 44.2% of the patient group. Some researchers have pointed out that, compared with males, females have better OI ability (24). Third, different methods were used to measure olfaction in different studies. The UPSIT was used in Hardy et al.’s study (16), while the SST was used in the current study to measure OI function. Finally, the inclusion criteria and clinical symptom assessment methods differed between the studies. In Hardy et al.’s study, the criterion for the remission period of bipolar mania was YMRS score ≤ 9, and the factor analysis of the PANSS scale was used to assess depression and anxiety symptoms (16), whereas the inclusion criteria for the remission period of bipolar mania in the current study was YMRS ≤7, and the HAMD scale was used to assess depressive symptoms.

In our study, the olfactory function of the episodic BD, euthymic BD, and HC groups were compared in pairs. The episodic BD group showed obvious impairment of OS and OI, while the OI of euthymic BD group was still impaired, and there was no obvious difference between OS in remission and the HC group. This finding suggests that OI may be a trait marker of BD but is not specific, while OS may be a state marker that can be used to distinguish between the episodic and euthymic stage. Previous studies support this result. Li et al. (4) proposed that OI defects may still exist in BD patients after remission, and that OS will recover with the improvement of the condition. Kazour et al. (18) divided 176 participants into five groups: a bipolar depression group (*n* = 33), a euthymic BD group (*n* = 30), a unipolar depression group (*n* = 33), a unipolar euthymic group (*n* = 31), and an HC group (*n* = 49) for olfactory ability. The results revealed no difference in OS between the euthymic BD and HC groups, which was consistent with the results of our study. However, their results indicated that OI and OS in patients with bipolar depression were not significantly impaired compared with HCs, and OI in the euthymic BD group was not impaired (18). This conclusion is not consistent with the results of our study. One possible reason is that Kazour et al.’s study was a cross-sectional study comparing olfactory function among different groups, whereas a more accurate assessment would be a longitudinal comparison of the same patients in the episode and remission of bipolar depression. Second, the smoking rate in the episodic group in Kazour et al.’s study was 48.5%, the smoking rate in the euthymic group was 50%, and the smoking rate in the HC group was 42.9%, whereas the smoking rate in the episodic group in our study was 25.7%, the smoking rate in the euthymic group was only 26.9%, and the smoking rate in the HC group was only 13.6%. McLean et al. (26) reported that smokers in the patient group scored higher on the UPSIT test than non-smokers, while smokers in the HC group scored lower than non-smokers, suggesting that smoking may “normalize” OI in some patients with mental disorders. Third, the inclusion criteria for patients in remission in Kazour et al.’s study required that patients were in remission for at least 3 months and scored less than 9 points on the Montgomery Depression Rating Scale, which differed from the inclusion criteria and evaluation methods for patients in the episodic and euthymic periods in our study. Fourth, Kazour et al.’s study recruited far more female than male participants. Kazour et al. (19) first compared olfactory function in a group of bipolar manic patients, euthymic BD patients, and HCs, revealing that, compared with healthy controls, OI was impaired during the episodic and euthymic period. This is consistent with the results of our study. However, Kazour et al. (19) reported that there were no significant differences in OS among the three groups, which differs from the results of our study. Possible reasons include the younger average age of patients in bipolar manic in Kazour et al.’s study (28.4 years old), the higher proportion of smokers in each group, and differences in the inclusion criteria. However, our study examined a broader sample that included not only bipolar manic patients, but also patients with bipolar depression.

### 4.3. Correlation between olfactory function, emotional symptoms, and social function in BD

The current findings revealed that the OS of episodic BD was inversely correlated with HAMA scores; the more severe the anxiety of episodic BD patients, the lower the OS. Additionally, clinical studies have reported that BD is often accompanied by anxiety disorders. Hardy et al.’s study (16) indicated that more severe fear and avoidance behavior are associated with worse OS, and anxiety symptoms are associated with decreased OS function, consistent with the results of the current study. However, Clepce et al. (27) compared the olfactory function of 17 patients with anxiety disorders and 17 HCs, and the results revealed no significant differences in OS and OI between the patient group and the HC group. This conclusion is inconsistent with the results of the current study, possibly because the study targeted patients with anxiety disorders, including phobias, obsessive-compulsive disorder, and generalized anxiety disorder, while our study enrolled patients with BD with anxiety symptoms.

Our study revealed that the OS score of patients with episodic BD was negatively correlated with PANSS score; the more severe the psychiatric symptoms of patients, the lower the OS. When we divided the patients into the P-BD and NP-BD groups according to whether BD was accompanied by psychotic symptoms, our results revealed that the OS score of episodic NP-BD patients was negatively correlated with PANSS scores. Corcoran et al. (28) noted that OI impairment was associated with negative symptom severity, but not with positive symptom severity or general psychopathological symptoms. Good et al. (29) also reported that negative symptoms were associated with significantly lower UPSIT scores when assessing olfactory function in patients with schizophrenia. The results of the previous studies mentioned above differed from the current findings, possibly because of factors such as the source of the study subjects and the different methods used for psychological scale assessment. Further research should be carried out to clarify the correlation between olfactory function and psychiatric symptoms.

In the current study, OS and OI tests were performed on euthymic BD patients. The results revealed that the correlations between OS and OI scores, emotional symptoms, and social functions in the euthymic group was not statistically significant. However, when we divided BD patients into P-BD and NP-BD groups, we found that the OS scores of patients in the euthymic NP-BD group were negatively correlated with HAMD scores, which is consistent with the depressive syndrome of depressed patients. The main clinical manifestations of depressive syndrome are low mood, lack of interest and loss of fun. A study by Li et al. (4) also supports the current results, suggesting that OS in patients with bipolar depression is negatively correlated with HAMD scores. Henry et al. (2) reported that olfactory disorders affect quality of life and are associated with severe depressive symptoms. Hardy et al. (16) examined the correlation between olfactory function and mood symptoms in euthymic BD patients, reporting that increased OS was associated with depressive symptoms. Inconsistencies in results between studies may be related to differences in the sample sizes, scale assessment tools, and olfactory testing methods in different studies. To date, few relevant studies have investigated olfactory function, emotional symptoms, and social function, and it will be necessary to carry out relevant studies with larger case sample sizes, more objective olfactory testing methods, and better reliability and validity of the emotional and social function assessment scale to further confirm the correlation between olfactory function, and psychological symptoms, and social function in BD patients.

### 4.4. Correlation between olfactory function and demographic characteristics in BD patients

The current study explored the correlation between olfactory function and demographic characteristics in episodic and euthymic BD patients, and revealed a correlation between olfactory function and some demographic characteristics in BD patients. Specifically, OI in all episodic BD patients was negatively correlated with the course of the disease and positively correlated with the number of years of education. The OS of NP-BD patients was negatively correlated with age and the course of the disease. The OI of NP-BD patients was positively correlated with years of education, and sex was associated with OI. The OS and OI of in euthymic NP-BD patients were positively correlated with the years of education.

Previous studies have reported differences in olfactory function in different age groups, and age-related decreases in olfactory function. Attems et al. (30) reported that 50% of people aged 65–80 years exhibit olfactory function decline. Older people exhibit increased rates of cognitive function and memory impairment, and OI ability impairment is associated with cognitive and memory decline. This may explain why olfactory impairment becomes more pronounced with age. Ship and Weiffenbach (31) proposed a correlation between age, sex, and olfactory function. Sorokowski et al. (32) compared the differences in olfactory function between men and women. The results were similar to those in the current study, indicating that women’s olfactory ability is better than that of men. However, some researchers have reported that there is no correlation between sex and OI (8). Possible reasons for the inconsistency between studies include the small sample size, large heterogeneity of cases, and the influence of confounding factors that are not completely excluded. At the same time, some researchers pointed out that there is a correlation between the years of education and OI and OS (4). Fornazieri et al. (33) controlled for confounding factors such as age, sex, and smoking, and UPSIT scores (i.e., OI) were still positively correlated with education level. To date, few studies have examined the relationships between olfactory function and demographic characteristics of BD patients, and more studies will be needed to confirm the current results in future.

## 5. Limitations

The current study involved several limitations. First, the size of the sample in our study was small, and the follow-up had a high shedding rate. In addition, our study did not distinguish emotional episodes types. Most of the enrolled patients were bipolar manic, while some had bipolar depression. Thus, further studies will be required to evaluate the olfactory function of BD patients with different emotional episodes in a more detailed manner. Second, the olfactory evaluation adopted in this study is a relatively subjective detection method, which is easily affected by cultural differences, living environment and other factors, and the results will inevitably produce some errors. In the future, we will employ more objective methods for detecting olfactory function, such as olfactory event-related potentials, which can be considered to minimize errors caused by subjective experience. Third, patients in the case group were treated with psychotropic drugs, and different types and doses of drugs may have different effects on olfactory function, thus affecting the accuracy of the results of this study. More rigorous prospective controlled studies should be conducted in the future to explore the effects of different types and doses of drug treatment on olfactory function. Finally, the clinical data collected in our study, including the course of the disease, and age to first onset, may have been affected by the patient’s recall bias. This impact can occur even though we ask patients or families to provide original medical records whenever possible, or we consult hospital records to verify relevant information.

## 6. Conclusion

Patients with episodic BD have impaired OS and OI, and are not associated with psychotic symptoms. OI in euthymic BD patients is still impaired, and OS in remission is not significantly different from that of HCs. The results indicate that OI may be a characteristic marker of BD, and OS may be a state marker that can be used to distinguish between the episodic and euthymic stages of BD. More rigorous studies are needed in the future to confirm these results.

## Data availability statement

The original contributions presented in the study are included in the article/supplementary material, further inquiries can be directed to the corresponding authors.

## Ethics statement

The studies involving humans were approved by the Ethics Committee of Shunde WuZhongpei Memorial Hospital, Foshan City, China. The studies were conducted in accordance with the local legislation and institutional requirements. The participants provided their written informed consent to participate in this study.

## Author contributions

YL: Conceptualization, Methodology, Data curation, Writing – original draft, Investigation, Formal analysis. HY: Data curation, Writing – original draft, Investigation. XL: Data curation, Writing – original draft, Investigation. LS: Data curation, Writing – original draft, Investigation. CY: Data curation, Writing – original draft, Investigation. CC: Conceptualization, Methodology, Supervision, Writing – review & editing. CL: Conceptualization, Methodology, Project administration, Supervision, Writing – review & editing.
